# Redesigning Death Rounds: Alleviating distress for residents in end-of-life care

**DOI:** 10.12688/mep.20212.2

**Published:** 2025-01-13

**Authors:** Shannon Fang, Lauren Baumgardner, Benjamin Schwan, Vidya Krishnan

**Affiliations:** 1 School of Medicine, Case Western Reserve University, Cleveland, Ohio, 44106, USA; 2 Department of Palliative Care and Hospice, The University of North Carolina at Chapel Hill, Chapel Hill, North Carolina, 27514, USA; 3 Center for Biomedical Ethics, The MetroHealth System, Cleveland, Ohio, 44109, USA; 4 Department of Pulmonary, Sleep, and Critical Care, The MetroHealth System, Cleveland, Ohio, 44109, USA

**Keywords:** Death and dying, palliative care, continuing education, collaborative/peer-to-peer, integrated curriculum, terminal care, internship and residency, psychological well-being

## Abstract

**Introduction:**

Residents report limited end-of-life care training, resulting in negative socio-emotional impacts, burnout, and inadequate patient care. An academic urban county hospital adopted the Death Rounds (DR) conference for residents in the medical intensive care unit as a monthly free-form discussion to help residents cope with the emotional aspects of caring for dying patients. Our goal was to implement and evaluate a newly structured DR curriculum to help residents further reflect on experiences of caring for dying patients, reduce emotional burnout, and improve physician well-being.

**Methods:**

Using a mixed-methods design, we conducted a qualitative needs assessment using interviews of residents. DR conference modifications based on the needs assessment include shorter, more frequent sessions; breakout groups; prompts for facilitating discussion; and multidisciplinary facilitators. A pre-post modification survey using the Likert scale was administered to all residents to assess the programmatic changes.

**Results:**

Pre- and post-modification data were received from 30 and 50 of 116 residents, respectively. A greater proportion of post-test DR attendees reported that DR helped them feel less distressed when caring for dying patients (p=0.018). Among residents who did not attend DR, there was greater agreement in feeling emotionally supported by their team when caring for dying patients (p=0.046). Overall, 81% of post-test respondents agreed DR was worthwhile of their time, and almost all respondents agreed discussing the emotional impacts of patient death is important.

**Conclusion:**

Death Rounds is a replicable and impactful curriculum that helps residents process the challenges of caring for dying patients and may improve emotional distress and team support.

## Introduction

Residents often report limited preparation and training to cope with the emotional and psychological impacts of caring for dying patients ^1^. Feelings of failure, helplessness, and guilt are common reactions to patient death, and without proper support, can lead to negative socio-emotional consequences, burnout, and depersonalized patient care ^2– 4^. This curricular gap is particularly important, as physicians are increasingly involved in the care of dying patients due to advances in life-saving and life-prolonging technology ^5^.

Current graduate medical education has limited formal opportunities to address physician grief and distress, and there is limited evidence supporting the available interventions and impacts that do exist. Our study expands on the current literature regarding the effectiveness of Death Rounds (DR), a monthly one-hour conference with residents in the Medical Intensive Care Unit (MICU) ^6^. These sessions consist of open-ended discussions facilitated by multidisciplinary faculty to help residents process their thoughts and emotions about caring for dying patients. The implementation and evaluation of DR has not been replicated in the literature to date. Although similar programs have been established, many of these curricula are focused on students or singular workshops rather than integrated curricula for residents ^7, 8^. Simulations and multi-hour workshops are also resource- and time-intensive ^7, 9, 10^. However, DR utilizes few resources, does not require special training or preparation, and has limited time demand.

Our study aims to improve the Death Rounds curricula and evaluate the impact of Death Rounds on residents in the MICU. We predict that introducing structured guidance into the free-form Death Rounds sessions will guide residents toward a more impactful and reflective conversation to help cope with caring for dying patients, promoting resident well-being and quality end-of-life care for patients.

## Methods

### Settings and participants

Death Rounds was implemented in 2016 at an urban, academic hospital. All medical students and residents rotating in the MICU were welcome to attend, primarily internal medicine (IM) residents and medicine-pediatrics (MP) residents, as well as rotators from other disciplines such as emergency medicine, family practice, and maternal-fetal medicine fellows.

### Intervention

Death Rounds is a monthly, one-hour free form discussion that takes place in the MICU and is facilitated by intensive care, palliative care, ethics, and pastoral care faculty ^11^. Sessions begin with the distribution of a list of patients who died in the MICU during the past month. Participants are then invited to openly share their experience with a patient and ask questions. DR focuses on personal, social, emotional, or ethical reflections, rather than medical questions. DR attendance is not required but open to all trainees and providers.

We conducted a qualitative needs assessment in July 2022 of Death Rounds using phone call interviews with a representative sample of 14 IM and MP residents who were individually selected to provide a breadth of interest and experience with DR. The interviews were semi-structured using a list of seven questions regarding the strengths, weaknesses, and goals of the current program. Thematic analysis was completed on the interviews, with a focus on how to guide programmatic changes to help improve the experience and effectiveness of DR. The DR modifications implemented based on the needs assessment are outlined in Table 1.

**Table 1.** Death Rounds modifications implemented based on the qualitative needs assessment.

Modification	Reason
Shorter sessions: 30 minutes instead of 1 hour	Residents reported limited availability in their schedules.
Increased frequency: biweekly instead of monthly	Residents reported desire to discuss patients closer to their time of death.
Breaking out into small groups	Residents reported greater comfort sharing personal insights with a smaller group of peers.
Utilizing a prompt card to facilitate discussion	Residents reported uncertainty about how or what to contribute to the discussion.
Incentives for participation, including attendance points and refreshments	Recognition of time and effort dedicated to DR.

The prompt card modeled off Eng *et al*. ^12^, included questions to stimulate discussion and reflection if needed, including asking what was difficult about the patient’s death, how the death impacted the resident, and how the resident managed their emotional response to the death. Utilization of this facilitation card helped to encourage reflective conversation without the need for formal training ^9, 10^.

Strengths that were continued include using the setting of a quiet team room and engaging multidisciplinary facilitators (palliative care, ethics, pastoral care, and critical care) to learn about end-of-life care from various specialties. The final DR agenda included 5 minutes of check-in, 20 minutes of small-group breakouts for reflection with prompt cards, and 5 minutes to regroup for check-out and learning points.

### Survey

A pre-modification and post-modification survey was administered in July 2022 and February 2023, respectively, to all IM and MP residents to assess the programmatic changes, regardless of their attendance at DR. The survey was administered online through email, including up to three reminder emails. The post-modification survey was administered 6 months following implementation of the curricular changes. The survey consisted of questions about training experience and experience with patient death, as well as 10 questions related to values (1), coping (1), self-awareness (2), support (2), distress (2), and clinical comfort (2) using a Likert scale. The Likert scale ranged from 1 (“strongly disagree”) to 5 (“strongly agree”).

A second set of similar questions were completed by those who indicated they had attended at least one Death Rounds. The second set of questions asked the attendees if Death Rounds helped them improve in the aforementioned areas using the 5-point Likert scale. As an example, if the question in set one is “I feel distressed when caring for dying patients,” then the question in set two is “Death Rounds helped me feel less distressed when caring for dying patients.” Utilizing both sets of questions allowed us to assess the direct impacts of DR and the modifications, as well as compare responses to residents who did not attend DR. Two free-response questions were included at the end of the survey.

This study was approved for exemption by MetroHealth’s Institutional Review Board (STUDY00000153) on 7/14/2022 because the research only includes interactions involving survey procedures. Written informed consent was obtained from all participants.

### Data analysis

Data analysis was performed in three separate survey groups: DR attendees (intervention group), DR non-attendees (non-intervention group), and DR-specific questions. Pre-test DR attendees include participants who attended at least one DR before curricular modification; post-test DR attendees include participants who attended at least one DR after curricular modifications. DR non-attendees include participants who have never attended Death Rounds before or after modifications. The DR-specific questions include the second set of survey questions only completed by DR attendees.

Pre- and post-Likert scale responses were analyzed using a two-sided Fisher exact test at a significance level of p=0.05 in each population. Agreement rates, defined as the percent of participants who answered “strongly agree” or “agree”, for each question were used to compare pre- and post- DR modification responses.

## Results

Pre-modification data were received from 32 of 116 residents, and 6-month post-modification data were received from 50 of 116 residents. All levels of training are represented in our survey, as well as internal medicine and medicine-pediatric residents ( Table 2). Pre-test and post-test respondent characteristics were similar, including the median number of patients participants had been with at time of death.

**Table 2.** Survey participant characteristics.

	Pre-test [N=32]		Post-test [N=50]	
	Attended DR [n=9]	Not attend DR [n=23]	Attended DR [n=21]	Not attend DR [n=29]
PGY-1	3 (33%)	8 (34%)	2 (10%)	14 (48%)
PGY-2	1 (11%)	6 (26%)	9 (43%)	5 (17%)
PGY-3	4 (44%)	7 (30%)	10 (47%)	8 (28%)
PGY-4	1 (11%)	2 (9%)	0 (0%)	2 (7%)
Internal medicine resident or preliminary resident	3	21	18	26
Medicine-pediatrics resident	6	2	3	3
Median number of patients been with at time of death	6–10	1–5	6–10	1–5

**Figure 1.**
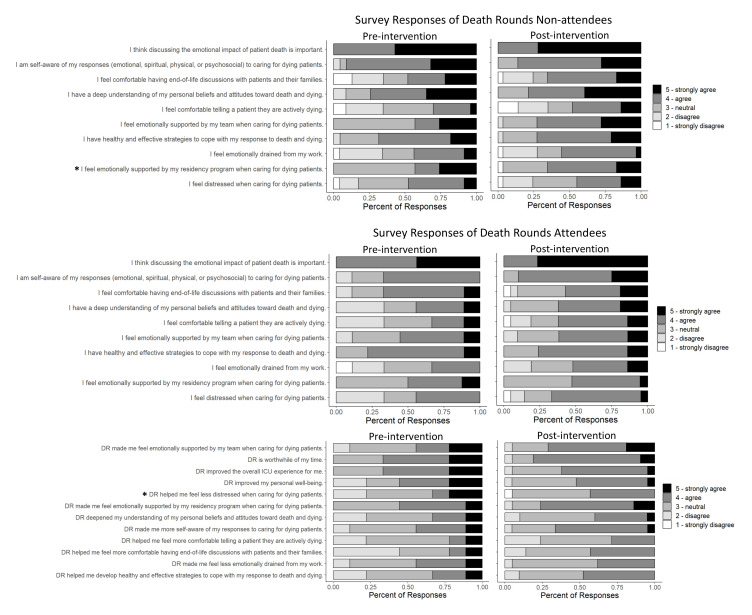
Summary of responses to the pre-intervention and post-intervention surveys. Data is stratified by those who did not attend DR and who attended ≥1 DR. Stars indicate questions with a significant difference in pre-test and post-test responses (p-value <0.05).

Responses to the survey statements are summarized in Figure 1. The majority of residents reported feeling distressed when caring for dying patients and emotionally drained by their work. Nearly all residents agreed discussing the emotional impact of patient death is important.

Among DR non-attendees, there was a significant difference between pre-test and post-test responses to the question about team emotional support when caring for dying patients (p=0.046). Approximately 43% of pre-test respondents agreed they felt supported by their team, compared to 73% of post-test respondents. This increased feeling of support among DR non-attendees may indicate DR helps build team emotional support, even for those who did not attend the conference.

Responses to DR-specific survey statements are also summarized at the bottom of Figure 1. Among DR attendees, there was a significant difference in the question regarding DR helping residents feel less distressed when caring for dying patients. Compared to the pre-test, post-test DR attendees overall reported that DR helped them feel less distressed when caring for dying patients (p=0.018), with a 10% increase in agreement.

Increases in agreement were also seen in statements regarding DR helping with self-awareness (44% to 67%), emotional support (44% to 71%), and comfort with end-of-life conversations (22% to 43%), though not statistically significant. Overall, less than half of participants agreed that DR specifically helped them develop coping strategies, deepen their understanding and beliefs toward death, feel less emotionally drained, and feel comfortable telling a patient they are actively dying. DR can continue to improve, with a focus on these areas and outcomes.

About half of participants reported DR improved their personal wellbeing, and a little over 60% agreed DR improved the overall MICU experience. Residents reported a 14% increase (67% to 81%) in agreement that DR is a worthwhile use of their time.

## Discussion

Our study supports that ICU residents overwhelmingly believe discussing the emotional impact of caring for dying patients is important, and Death Rounds is a worthwhile curriculum for residents to address and process these impacts. Residents also report feeling increasingly distressed when caring for dying patients and emotionally drained by their work. Creating and evaluating a Death Rounds conference is replicable across institutions to help residents cope with caring for dying patients and may improve self-awareness, team support, and distress.

Patient death can precipitate negative emotional and psychosocial effects on physicians, including burnout, depersonalization, and inefficacy ^13^. The lack of education around physician grief, in addition to the fast-paced work environment, creates a harmful culture of emotional suppression in the workplace ^14, 15^. Lectures inadequately teach how to manage the personal impacts of caring for a dying patient, and trainees instead value reflection and debriefing on their lived experiences of caring for dying patients ^15– 17^. We believe adding a structured framework to Death Rounds, including small-group breakouts and facilitation cards, helped promote formative reflections on lived experiences in patient care.

Our study aligns with previously published findings on curricula similar to DR and contributes to the growing body of literature suggesting that DR provides a beneficial and meaningful experience for ICU residents. One initial study reported that residents believed DR should be integrated into all ICU rotations, as it improved the overall ICU experience ^6^. These results were consistent with findings from the implementation of DR in a neurology inpatient unit, where it provided an emotional outlet and valuable education on end-of-life care ^18^. Similarly, a Patient Death Debriefing Session program conducted during a medical oncology rotation demonstrated improved resident perceptions of emotional support and coping, which was also demonstrated by our findings ^12^. These services often involve higher rates of patient death and may be more likely to benefit from the impacts of DR. While our sample size was insufficient to allow for comparisons, prior research indicates that higher attendance at DR sessions is associated with improved ability to cope with the emotional challenges of caring for dying patients ^6– 12^.

Several recurring themes emerged during DR discussions, including guilt, insecurity, and uncertainty. Residents frequently expressed feelings of guilt, particularly regarding their perception of having not done enough to save the patient. Residents also shared their apprehension in effectively communicating with patients and their families regarding the dying process, including challenges with helping families comprehend the severity of the patient’s condition. Uncertainty was prominent when residents navigated conflicts between their personal assessment and the family’s assessment of the patient’s best interests.

Resident free-responses showed that DR has been useful for “getting feelings out.” One free-response answer wrote: “It can be very difficult to deal with these emotions especially when still in duty. I feel that further discussion of these emotions and possibly coping mechanisms can really make a drastic difference.” DR can also help build a more open culture of vulnerability and connection: “Often I find myself holding a lot of emotions in and then it re-surfaces at random times in the future. It would be nice to know that other colleagues might be processing through these difficult cases too.” DR is not required by all residents, as some residents noted they prefer to process and receive support through other means.

Based on our findings, DR may help residents feel less distressed when caring for dying patients. Reflective practice can enhance a resident’s personal awareness of reactions to caring for dying patients, which can then guide management and coping strategies. This process translates to greater personal and professional wellness and quality patient care ^13, 19– 21^. Residents who did not attend DR reported an increase in post-test agreement about feeling emotionally supported by their team when caring for a dying patient. This data suggests DR may contribute to improving overall team emotional support, even for residents who did not attend the conference.

Notably, DR is adaptable across various clinical settings and medical specialties due to its flexibility in session frequency, duration, and location. Facilitators do not require specialized training, as the use of prompt cards can effectively guide discussions. Furthermore, the implementation of DR does not necessitate additional funding, making it a practical and accessible curricula for residents.

Our study has several important limitations. First, the survey was completed by less than half of the residents, so the data may not be representative of all residents. Residents who had greater interest in palliative care or end-of-life care may have been more likely to complete the survey and attend Death Rounds. This selection bias may overestimate the value and benefits of DR. Second, the survey was not tested in this population or validated. Due to the number of questions in our survey, the significant findings may be due to chance from multiple comparisons and may not reflect true differences. Third, our sample size was relatively small at a single site, limiting our ability to detect significant statistical associations.

Gaps in the DR curricula identified in our study include helping residents develop coping strategies, feel less emotionally drained, and feel comfortable telling a patient they are actively dying. Future studies include further evaluation of impact on patient care, long-term follow-up with residents, and multi-institution studies.

## Data Availability

Figshare: Death Rounds Pre-post Survey Data. https://doi.org/10.5281/zenodo.10615622 ^22^ This project contains the following underlying data: Zenodo data.xlsx. Figshare: Death Rounds Pre-post Survey Data. https://doi.org/10.5281/zenodo.10615622 ^22^ This project contains the following extended data: A prompt card to facilitate discussion.pptx Data are available under the terms of the Creative Commons Attribution 4.0 International license (CC-BY 4.0).
