# Supplementary material for: Redesigning Death Rounds: Alleviating distress for residents in end-of-life care
Source: MedEdPublish (2016). 2025 Jan 13;14:31. Originally published 2024 May 3. [Version 2] doi: 10.12688/mep.20212.2 (PMC11782931; doi:10.12688/mep.20212.2)
Supplement: Peer review history

# Reviewer report: Reviewer response for version 2

**DOI:** 10.21956/mep.22336.r40969

**Published:** 2025-01-30

**Authors:** Barbara Jennings

**Affiliations:** 1 University of East Anglia, Norwich, England, UK

I learned a lot about the educational rationale and principles of using death round conferences (DR) as part of continuous professional development for residents in hospital medicine by reading this well-written article.

 The authors describe an implementation and evaluation study, this is version 2 of their manuscript, and it provides all I would expect of a final research paper. And because of the open peer review, I can see the authors’ account of all changes made to develop the article between versions 1 and 2.

 I found clear descriptions of research governance and the study setting; open access to evaluation tools (prompt card for interviews) and full data sets via figshare links; and a full exploration of the studies strengths and limitations in the discussion.

 The introduction explains the educational intervention and compares the use of monthly DRs for resident doctors with alternative approaches such as simulation and workshops.

 The methods section described a mixed qualitative and quantitative approach, and the aims spanned evaluation and educational improvement. The needs assessment, semi-structured interviews and analysis of data are systematically described.

 For the results, the layout of tables and figures with descriptive titles and legend (for the graphical presentation of survey data) is a real strength within a well-presented paper.

 Finally, the authors explore the significance and any limitations (linked to selection bias that most evaluation studies are at risk of) to their study design and findings in an interesting discussion. The benefits with respect to personal and professional wellness are important findings.

 I think this article will be of interest to most clinical educators and postgraduate supervisors as well as specialists in this area, such as oncologists and those working in emergency medicine and intensive care.

 Thank you to the team for sharing their important project and findings.

Have any limitations of the research been acknowledged?

Yes

Is the work clearly and accurately presented and does it cite the current literature?

Yes

If applicable, is the statistical analysis and its interpretation appropriate?

Yes

Are all the source data underlying the results available to ensure full reproducibility?

Yes

Is the study design appropriate and is the work technically sound?

Yes

Are the conclusions drawn adequately supported by the results?

Yes

Are sufficient details of methods and analysis provided to allow replication by others?

Yes

Reviewer Expertise:

Medical education, faculty development, life sciences

I confirm that I have read this submission and believe that I have an appropriate level of expertise to confirm that it is of an acceptable scientific standard.

# Reviewer report: Reviewer response for version 2

**DOI:** 10.21956/mep.22336.r40896

**Published:** 2025-01-21

**Authors:** Tim Lahey

**Affiliations:** 1 University of Vermont Medical Center, Burlington, VT, USA

I commend the authors for doing an excellent job responding to prior feedback. Their data remains of interest to educators, and is now accompanied by study limitations. I hope this will help more residents have an outlet for discussion of difficult cases.

Have any limitations of the research been acknowledged?

Partly

Is the work clearly and accurately presented and does it cite the current literature?

Yes

If applicable, is the statistical analysis and its interpretation appropriate?

Partly

Are all the source data underlying the results available to ensure full reproducibility?

Yes

Is the study design appropriate and is the work technically sound?

Partly

Are the conclusions drawn adequately supported by the results?

Partly

Are sufficient details of methods and analysis provided to allow replication by others?

Partly

Reviewer Expertise:

Epidemiology, ethics, medical education

I confirm that I have read this submission and believe that I have an appropriate level of expertise to confirm that it is of an acceptable scientific standard.

# Reviewer report: Reviewer response for version 1

**DOI:** 10.21956/mep.21638.r36947

**Published:** 2024-05-14

**Authors:** Tim Lahey

**Affiliations:** 1 University of Vermont Medical Center, Burlington, VT, USA

Fang et al present a well-written description of a modified approach to death rounds (DR), an established educational and well-being intervention for trainees managing moribund patients.

 Importantly, the authors replicate the prior finding that residents found DR to be helpful. That alone is useful information.

 In their attempt to extend their findings into an assessment of how a changed format of DR affected resident perceptions, however, the authors run into methodological problems.

 Specifically, their study design is not unreasonable, but it has important limitations that the authors only partly acknowledge. The study results are well-described, but the authors' interpretation a bit too confident given the potential sources of bias introduced by this study structure issue. 

 In more detail, the authors' present pre/post-intervention survey results using data from two distinct groups of residents. Pre-intervention, 32 residents responded, whereas 50 residents responded post-intervention. The percentage of residents who actually attended death rounds varied between those groups. These pre/post-intervention group differences raise the possibility that differences in pre/post-intervention responses are the result not of the interval intervention but rather intrinsic differences in these different groups of residents' responses to end-of-life care. This is particularly a problem since - the only piece of this source of bias the authors acknowledge - reactions to end of life care may have influenced the likelihood of survey response.

 The authors attempted to address this source of bias, at least partly, by presenting survey response data "stratified by those who did not attend DR and who attended ≥1 DR." This is helpful, but demographic data allowing reader to assess comparability of respondents who did attend pre/post-intervention are not provided nor are the sizes of the different respondent groups. That leaves the reader unable to assess how much this source of bias has truly been mitigated.

 In addition to presenting stratified data more effectively, with number of respondents and demographic data by attendance status, the authors should acknowledge the implications of this source of bias more clearly in their discussion. Did greater number of DR attended correlate with likelihood of feeling more comfortable in end-of-life care before or after the survey?

 In addition, while the authors' interpretations focus on the couple of questions that yielded statistically significant differences in pre vs post intervention responses, they do not mention that this finding could be the result of chance due to conducting multiple comparisons. By comparing more than 30 question responses pre/post-intervention, it's unsurprising that 1 or 2 would yield a significant difference just by chance. The authors did not correct for multiple comparisons in their approach to statistical analyses nor mention this issue in their discussion. They should do at least the latter.

 My suggestion would be to focus on replicability of DR, and the interesting characterization of residents' experience of end-of-life care, and then presentation of pre/post-intervention data with caveats related to sources of bias or the play of chance more appropriately foregrounded. The authors' contention that, "DR most significantly helped residents feel less distressed when caring for dying patients" and "DR may contribute to improving overall team emotional support, even for residents who did not attend the conference" or the suggestion that DR had a role in "helping residents develop coping strategies, feel less emotionally drained, and feel comfortable telling a patient they are actively dying" are not fully justified by the data and should be expressed more cautiously.

 Additional points below are aimed at improving manuscript clarity, which is generally high.

 1. Introduction. The first sentence of the second paragraph, beginning "Current graduate medical education...", is a little garbled, and somewhat redundant given the foregoing paragraph. The sentence can be improved by removing the initial phrase, and starting with "There is limited evidence regarding interventions that ameliorate deleterious resident responses to end-of-life care," or something analogous.

 2. Methods. In the "Intervention" section, the authors describe DR as an intervention that lasts one hour - as described in the original reference, #6 - but then most of their results concern an altered, briefer format. That shift might lead to reader confusion which could be avoided by characterizing what DR is, intrinsically, without reference to the hour, and then subsequently point to flexible traits like duration that were changed. In the subsequent paragraph, the authors should characterize how the 14 residents who responded to their 7-question needs assessment were chosen.

 3. Results. The authors should ensure agreement of verbs linked to the plural noun "data." For instance, the third word of the first sentence of the results should be "were."

Have any limitations of the research been acknowledged?

Partly

Is the work clearly and accurately presented and does it cite the current literature?

Yes

If applicable, is the statistical analysis and its interpretation appropriate?

Partly

Are all the source data underlying the results available to ensure full reproducibility?

Yes

Is the study design appropriate and is the work technically sound?

Partly

Are the conclusions drawn adequately supported by the results?

Partly

Are sufficient details of methods and analysis provided to allow replication by others?

Partly

Reviewer Expertise:

Epidemiology, ethics, medical education

I confirm that I have read this submission and believe that I have an appropriate level of expertise to state that I do not consider it to be of an acceptable scientific standard, for reasons outlined above.
